# A Synopsis of Signaling Crosstalk of Pericytes and Endothelial Cells in Salivary Gland

**DOI:** 10.3390/dj9120144

**Published:** 2021-12-01

**Authors:** Ioana Cucu, Mihnea Ioan Nicolescu

**Affiliations:** 1 Faculty of Medicine, “Carol Davila” University of Medicine and Pharmacy, 050474 Bucharest, Romania; ioana.cucu@stud.umfcd.ro; 2 Division of Histology, Faculty of Dental Medicine, “Carol Davila” University of Medicine and Pharmacy, 050474 Bucharest, Romania; 3 Laboratory of Radiobiology, “Victor Babeș” National Institute of Pathology, 050096 Bucharest, Romania

**Keywords:** regenerative medicine, angiogenesis, telocytes

## Abstract

The salivary gland (SG) microvasculature constitutes a dynamic cellular organization instrumental to preserving tissue stability and homeostasis. The interplay between pericytes (PCs) and endothelial cells (ECs) culminates as a key ingredient that coordinates the development, maturation, and integrity of vessel building blocks. PCs, as a variety of mesenchymal stem cells, enthrall in the field of regenerative medicine, supporting the notion of regeneration and repair. PC-EC interconnections are pivotal in the kinetic and intricate process of angiogenesis during both embryological and post-natal development. The disruption of this complex interlinkage corresponds to SG pathogenesis, including inflammation, autoimmune disorders (Sjögren’s syndrome), and tumorigenesis. Here, we provided a global portrayal of major signaling pathways between PCs and ECs that cooperate to enhance vascular steadiness through the synergistic interchange. Additionally, we delineated how the crosstalk among molecular networks affiliate to contribute to a malignant context. Additionally, within SG microarchitecture, telocytes and myoepithelial cells assemble a labyrinthine companionship, which together with PCs appear to synchronize the regenerative potential of parenchymal constituents. By underscoring the intricacy of signaling cascades within cellular latticework, this review sketched a perceptive basis for target-selective drugs to safeguard SG function.

## 1. Introduction

The salivary gland (SG) microvasculature represents a kinetic cellular system, crucial for maintaining tissue vitality and homeostasis. One of the key components of this complex are pericytes (PCs), mural cells that ensheath the abluminal interface of the endothelial lining, while sharing the same basement membrane (BM) [1,2,3]. The mutual interplay between PCs and endothelial cells (ECs) has come into view as central teamwork which governs the blood vessels maturation, remodeling, development, generation, and stabilization of new vessel-building blocks [2,4,5,6]. As a variety of mesenchymal stem cells (MSCs), PCs attract attention in the field of regenerative medicine by their pivotal commission in the matter of tissue regeneration and repair [7,8]. Correspondingly, PCs play a well-documented role in the dynamic and intricate process of angiogenesis, defined as the proliferation of ECs, sprouting, constitution, and branching of new vessels from pre-existing ones in order to establish interlinking capillary networks by mechanical support and paracrine factors [3,6,9,10,11,12,13,14]. Dysfunction of this comprehensive interconnection parallels the pathogenesis of SG disorders [15,16]. The absence of PCs is a hallmark of the disruption of vascular integrity [17]. In accordance, several diseases and stress conditions, such as inflammation and ischemia, autoimmune background (Sjögren’s syndrome), and post-radiotherapy in neoplastic contexture result in the disorganizing of SG microarchitecture [3,18,19]. These phenomena are ascribed to acinar cells atrophy, apoptosis, and uncontrolled progression of fibrosis and lead to SG dysfunction (hyposalivation, xerostomia), thereby exacerbating some of the pathological processes [20,21]. Of note, these disorders all correspond to inflammatory status emerging from microvascular dysfunction [18,22]. Restoring and safeguarding vascular integrity as the primary target might represent a gear in the “master plan” for the treatment of the above-mentioned pathologies [15,22]. Moreover, recent studies revealed the embryological origin of PCs (neuroectoderm-derived neural crest cells) in the head and neck regions, as demonstrated in chick-quail chimeras [16,23].

With the intention of discerning the traditional immunophenotype (CD146+/endoglin+/Platelet-derived growth factor receptor beta (PDGFRB+)) of PCs from other mesenchymal cells, a myriad of biomarkers have been identified [3,24]. It is paramount to emphasize that PCs constitute a heterogenous population with no specific marker, so their identification is contingent on a coalescence of multiple markers. One of the most used membrane-bound markers is NG2 (neural/glial antigen 2) [25], together with alanine aminopeptidase (CD13) and CD90 [2,25]. Another cytosolic marker useful in PCs identification is alpha-smooth muscle actin (alpha SMA) [26], related to modulating the blood flow [1,3]. In fact, these markers vary between organs and typify in distinct stages of development and in their pathophysiological circumstances. Communication between PCs and ECs is indispensable for the balance, homeostasis, and formation of the SG vasculature, which is synchronized with an array of signaling molecules that acts in a coordinated manner to manage several biological processes. This review highlighted a global picture of a complex interaction network of crosstalk among signaling pathways between PCs and ECs, expressing synergistic reciprocity that delineates a rational basis for different pathogenetic elements as therapeutic targets in SG diseases.

## 2. Pericytes and Their Relationship with Endothelial Cells

The SG vasculature is an indispensable complex of an accurately organized hierarchical tree of diversified cellularity, decisive in establishing and preserving tissue health by virtue of blood perfusion and through the potent reciprocity of the cells and the extracellular matrix (ECM). The build-up added to regeneration and/or repair of effective blood vessels turn out by intussusceptive angiogenesis, the chief event involving proliferation and migration of ECs and equilibrium of the microvascular lattice via coverage with PCs, archetypal multipotent stem cells (SCs), as a stringent fundamental ingredient [25]. The processes entailing ECs differentiation and organization into distinct miniatures are organotypic and tissue-specific and attach a note of intricacy to vascular development, balanced by shear stress and gene profile [27,28].

Within the framework of this complex organization, PCs are closely associated with endothelial coating, act as co-regulators of ECs, and are phenotypically divergent, being contingent upon the anatomical region [29]. On account of their perivascular location, Rouget cells were coined as PCs by Zimmermann, as reported by Brown et al. [30]. Transmission electron microscopy (TEM) upholds the identification of PCs features: they generally possess a large, spherical nucleus with profuse heterochromatin, a small amount of cytoplasm, incorporating collagenous and noncollagenous protein-synthesizing organelles with plenty of rough endoplasmic reticulum, in addition to a small proportion of glycogen particles, liposomes, and Golgi apparatus [12,31]. Moreover, PCs exhibit primary cytoplasmic finger-like projections disposed along the long axis of the vessel that give rise to orthometric secondary projections, linking to ECs [2]. Furthermore, microtubules take up the main part of all cytoplasmic processes, while intermediate filaments, including vimentin and desmin, are accumulated mainly within the primary ones [2]. The cytomorphological characteristics of PCs depend upon their differentiation circumstances, and also upon the anatomical site: they range from stereotypical flat and elongated to stellate shaped and are distinct in different zones of microcirculation [6,12].

**Figure 1:**
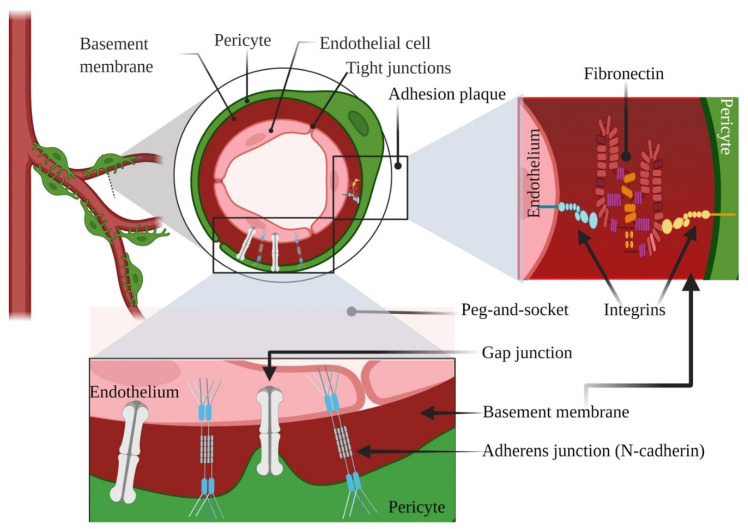
Illustrative representation showing typical pericyte-endothelial interconnections. Created with BioRender.com, Agreement number YY238VSLPL. Retrieved 26 November 2021.

According to our understanding, PCs exhibit two types of physical contacts with ECs: “peg-and-socket” and adhesion plaques [23]. Within the spots lacking a BM, cytoplasmic projections of PCs (the presumed “pegs”) liaise with ECs ‘ infoldings (the sockets), comprising N-cadherin-adherens junctions and gap junctions [2,8]. Upon some contact points, the dot-like adhesion plaques include principally fibronectin, connecting the plasma membrane with the BM through the intermediacy of the integrins [24]. A plethora of studies have suggested that the bidirectional signals between ECs and PCs point out the finale of vessel pliability and mark the quiescent level of vascular networks [32]. We illustrated briefly the typical pericyte-endothelial interconnections in Figure 1 as a starting point for the rest of our manuscript. Undoubtedly, proper communications are required for the maturation and stabilization of the SG vasculatures both in embryogenesis and adulthood, coordinated by a multitude of candidates and signaling molecules, such as Vascular endothelial growth factor (VEGF), WNT, NOTCH, HEDGEHOG (HH), Platelet-derived growth factor (PDGF), Transforming growth factor beta (TGFB), etc. [33,34]. Besides these biological mediators, extracellular vesicles (EVs), namely exosomes (EXOs), derived not only from PCs but also from ECs, which operate as information emissaries and stick out as potential biomarkers with promising therapeutic effects [35,36].

## 3. Changes in Salivary Glands Cell Repertoire

Although several pathological conditions including autoimmune diseases (Sjögren’s syndrome), irradiation, malignant tumors, and acute or chronic inflammation bring about disorders of the SGs, owing to the acinar cells hypofunction, and even apoptosis, all these pathologies exhibit corresponding microvascular dysfunction [37,38,39]. SG function is radically disrupted by radiotherapy (RT) as part of the multimodal treatment of head and neck cancer (HNC) [20]. Notwithstanding that SGs display slow-going proliferating tissue, the salivary acinar epithelial cells are highly sensitive to RT [40]. The emerging condition that evolves into hyposalivation, xerostomia/dry mouth, is accredited to monumental acinar cell irreversible loss, as well as to a lower likelihood of salivary gland progenitor cells (SGPCs) to differentiate into acinar cells [34,41,42].In an effort to explain the *modus operandi* of the radiation process with respect to puzzling, distinct radiosensitivity of salivary cells, Radfar et al. [21] noted that profiles of the irradiated parotid and submandibular gland were set apart by acinar atrophy, deprivation of translucent secretory granules, interstitial fibrosis, parenchymal loss, and duct proliferation. On the other hand, myoepithelial cells (MECs) engage in underpinning the morphogenesis and polarization of salivary acini [43]. Hakim et al. [44] found out that a significant post-irradiation (post-IR) loss of both alpha SMA and vimentin-positive MECs is coupled with a decrease of the proliferating rhythm in the acinar cells. Recently, the expression of CD44 was also reported as a marker of acinar cells in a MSCs population from the human parotid gland, along with PDGFRB and NG2, suggesting that these progenitor cell types could be PCs involved in the rescue of SG injury post-IR [3]. Along the same lines, the circulatory system is a requisite for conserving the viability and proper function of each adult organ. In this multiplex substructure, ECs are promptly subjected to death post-IR, and, therefore, targeting the disabled SG vasculature might be a successful appeal to prevent irreversible glandular damage [22,34]. Conditional upon slow turnover rate, within the vasculogenic zone, tissue-resident stromal cells, particularly PCs, hold up the matter of regeneration/repair [38]. There is a wealth of evidence illustrating that CD34-positive, CD31-negative adventitial SCs possess the capability to differentiate post-IR into PCs and encourage the new vessel generation by expressing *WNT* and *PDGF* genes, thereby supporting the idea that both blood vessel-framing ECs (CD34^+^CD31^+^ cells) and PCs may have a common mesenchymal precursor [45,46,47]. Interestingly, another recent study delineated that CD34-positive MSCs from the labial glands were virtually absent in patients suffering from Sjögren’s syndrome (SS) compared with healthy individuals [48].

Salivary gland tumors (SGTs) represent an assemble of histologically miscellaneous neoplasms that possess low prevalence among HNCs [49]. In the Hiroshima Tumor Tissue Registry, pleomorphic adenoma (PA) was the most frequent histotype and, subsequently, Warthin tumor and basal cell adenoma (BCA), whilst in the category of SG carcinomas are included adenoid cystic (ACC) and mucoepidermoid carcinoma [50]. Some SG carcinomas arise from dedifferentiation or else from benign tumors that develop into malignant ones [51]. Into the bargain, solitary fibrous tumor/hemangiopericytoma (SFT/HPC) constitutes a rare mesenchymal neoplasm; even so, in the WHO classification, the notion of HPC as a PC-derived tumor was cast aside on the side of fibroblast origin [52]. Even though the majority of SFTs follow a benign itinerary, a small percentage become malignant, or they can present zones of dedifferentiation of epithelial neoplasm mimicry [53]. De novo formation of SGTs, in addition to malignancy, is appointed to a plethora of signaling elements, the foremost hallmark being angiogenesis, evidenced through factors like VEGF and CD105 (endoglin) [54].

Crosstalks between PCs and ECs are acknowledged as culminate interlinkages in the labyrinthine marvelous process of angiogenesis. Straightforwardly, tumors surpass 2–3 mm^3^ and metastasize in the company of new vasculature that develops from five steps: (1) the increase of hypoxia-inducible factor 1-alpha HIF1Awhich attaches to hypoxia-response elements in the *VEGF* promoter that induces EC proliferation, followed by (2) the discharge of matrix metalloproteinases (MMPs) by both PCs and ECs, and degradation of the ECM/BM, (3) activation and migration of ECs, (4) formation of the capillary lumen, and ultimately (5) steadiness of tumor neovessels [6,55,56,57]. The stereotypical mechanism to assess the progression of angiogenesis is the analysis of microvessel density (MVD) within terms of units of vessels per high power fields (HPFs) employing immunohistochemical (IHC) methods to reveal certain EC-markers, aside from the well-known CD31 and CD34 and even greater CD105, the last one being substantiated as a fundamental co-receptor for the TGFB family, illustrating a pivotal role in angiogenesis [58,59].

## 4. Angiogenetic Behavior as a Consequence of PC/EC Crosstalk

Here, to make the landscape more complex, we reviewed the framework of elaborate protein-protein interrelatedness among decisive signaling pathways in a paracrine or autocrine manner. We underscored how the crosstalk intermingles to promote vascular development and the misregulations that can balance the homeostasis state into a tumorigenic program, as well as how the signaling components interface with each other to exert aversion to pharmacological approaches.

### 4.1. VEGF Signaling Pathway

VEGF is an assertive signaling factor that belongs to the PDGF supergene family and governs the angiogenic events during embryogenesis and adulthood in both pathological and physiological conditions [56,60]. The VEGF family is composed of five members: VEGF A-D and placenta growth factor (PGF) that interact with allied tyrosine kinases receptors (TKRs) VEGFR1-R3 [60,61,62]. VEGFA is the quintessential member of the VEGF family (referred to as VEGF henceforth), secreted by ECs in an autocrine loop and also produced by PCs as regards paracrine stimulation and binds to VEGFR1 and -2, expressed on the surface of ECs [10,60]. PCs also possess VEGFR1 on their surface, designated as a decoy receptor, and bind to VEGF, seizing it from ECs and deflecting the inauguration of angiogenesis, consequently promoting stabilization and quiescence in mature vessels [63]. In contrast, *VEGFR1* knockout leads to ECs hyperplasia and ectopic vascular morphogenesis [64]. The signal transduction through VEGFR2 is propagated intracellularly by plenty of downstream signaling pathways networks. Studies show that, dissimilar to most of TKRs which operate on RAS-RAF-mitogen-activated protein kinase (MEK), extracellular signal-regulated kinase (ERK) pathway, or Phosphatidylinositol 3-kinase (PI3K)/AKT/mammalian target of rapamycin (mTOR) pathway, VEGF/VEGFR2 highly activates the PLC-gamma-PKC-MAPK pathway, employed as the key indicator for EC proliferation [65,66,67,68]. Essentially, Growth factor receptor-bound protein 2 (GRB2) binds to the VEGF/VEGFR2 complex and associates with the Son of sevenless (SOS) to turn on RAS, which thereafter activates RAF that is competent to phosphorylate MEK and the last one further phosphorylates ERK1/2 [69,70,71]. Targeted deletion of *RAF*, *MEK1*, and *RAS-GAP* causes defective angiogenesis during embryogenesis [72,73,74]. Numerous studies suggest that RAS/RAF and after all ERK1/2 are fundamental for proper angiogenesis, ECs proliferation, survival, and motility [68,70,75,76]. Instead, compared with normal SG parenchyma, phosphorylated ERK1/2 immunoreactivity was increased in mucoepidermoid carcinoma samples by IHC; so, possibly, it can represent a therapeutic target for novel antitumor drugs [77]. Meanwhile, when HIF1A is upregulated and VEGF binds to VEGFR2 on normal ECs, and the RAS and PI3K pathways are set in motion [67]. PI3K is a dominant downstream effector pathway of RAS, which is instrumental in the formation of the normal blood vessel and ECs migration during angiogenesis [78,79,80]. When activated, PI3K converts phosphatidylinositol (4,5)-bisphosphate (PIP2) into phosphatidylinositol (3,4,5)-trisphosphate (PIP3), which sequentially binds to AKT/PKB, is expressed on ECs, and regulates a vast range of cellular responses [78,79]. AKT manages cell growth by entailing the phosphorylation of mTOR [67]. Markedly, The PI3K/AKT signaling pathway is upregulated in SGTs and hyperactivates, in part, mTOR, as a chief regulator of manifold cellular events such as cancer cell survival and metastasis [81,82,83]. PI3K signaling is usually intensified by the loss of function of the negative regulation of PTEN, whose main substrate is PIP3, thus enhancing the activation of AKT [84,85]. Correspondingly, several studies denote VEGF in respect to the tumor microenvironment (TME) as a prognostic factor commensurate with tumor size, aggressive behavior and metastasis, and cell growth [85,86,87]. Basically, TME is characterized by heterogeneous, aberrant vasculature derived from an imbalance among pro- and non-angiogenic factors [88,89]. Intriguingly, mTOR composes networks of crosstalk with the signaling pathways within the PI3K/AKT pathway [90] and the inhibition of mTOR determines the minimization of MVD and suppresses the tumor growth [82]. In addition to the chemotherapeutic drugs, such as bevacizumab and temozolomide, which inhibit VEGF in a paracrine loop and [10,91,92,93] provide the rationale to inhibit tumor progression, sorafenib, a multi-Tyrosine Kinase Inhibitor (mTKI), has been shown to restrict the action of VEGFR2, RAS kinase, and PDGFR, [94] targeting twofold PCs and ECs through the hindrance of the autocrine VEGF signaling loop [10].

**Figure 2:**
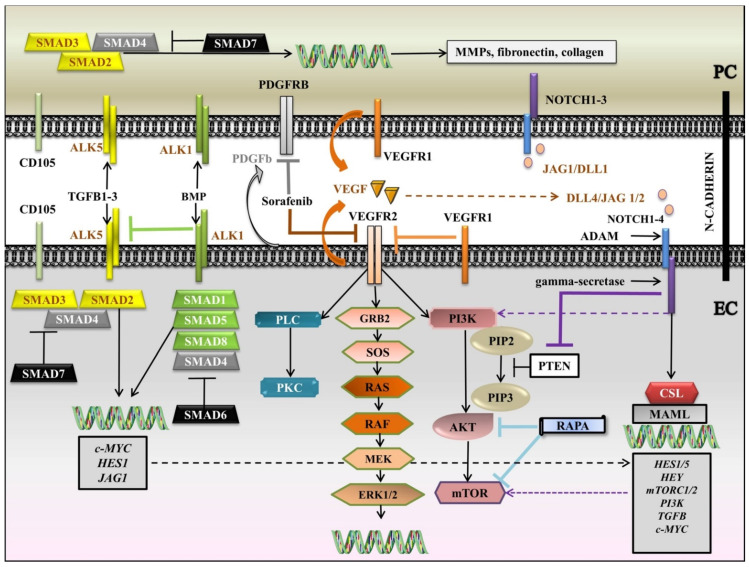
Diagram of the interlinking latticework among VEGF, NOTCH, PDGF, TGFB, and downstream signaling pathways. ADAM: Adisintegrin and metalloprotease; ALK: Activin-like kinase; BMP: Bone morphogenetic protein; CSL: CBF1/suppressor of hairless/LAG1; DLL: Delta-like; EC: endothelial cell; ERK1/2: Extracellular signal-regulated kinases 1/2; GRB2: Growth factor receptor-binding protein 2; JAG: Jagged; MAML: Mastermind-like; MEK: Mitogen-activated protein kinase kinase; MMPs: matrix metalloproteinases; mTKI: multi-Tyrosine Kinase Inhibitor; mTOR: mammalian target of rapamycin; NICD: Notch intracellular domain; PC: pericyte; PDGFB: Platelet-Derived Growth Factor b; PDGFRB: Platelet-Derived Growth Factor Receptor B; PI3K: Phosphatidylinositol 3-kinase; PIP2: phosphatidylinositol (4,5)-bisphosphate; PIP3: phosphatidylinositol (3,4,5)-trisphosphate; PKC: Protein kinase C; PLC: Phospholipase C gamma; RAPA: rapamycin; R- SMAD: receptor-regulated SMAD; SMAD: Small Mothers Against Decapentaplegic protein; SOS: Son of sevenless; TGFB: Transforming growth factor beta; VEGF: Vascular endothelial growth factor; black arrows: main signaling pathways; blunt-ended lines: blockade/inhibition; dashed arrows: induction/activation. Segments of the figure were sketched by using artworks from Servier Medical Art (15 November 2021). Servier Medical Art by Servier is licensed under a Creative Commons Attribution 3.0 Unported License, https://creativecommons.org/licenses/by/3.0/ (access on 15 November 2021).

We brought together the interlinking latticework among VEGF, NOTCH, PDGF, TGFB, and downstream signaling pathways in a diagram (Figure 2). Briefly, VEGF is secreted by both PC and EC and binds to VEGFR1, expressed by the two cell types, and VEGFR2, expressed by EC. VEGFR1 can enter a competition with VEGFR2 to seize VEGF from VEGFR2. Following the attaching of VEGF to VEGFR2, RAS-RAF-MEK-ERK and/or PI3K/AKT/mTOR and/or PLC-gamma-PKC pathways are set in motion. When actuated, PI3K adjusts PIP2 to PIP3, which further activates AKT and mTOR. In contrast, PIP3 is inhibited by PTEN. RAPA, another negative regulator, inhibits AKT and mTOR. NOTCH also plays a pivotal role in the regulation of angiogenesis. Both PC and EC display NOTCH 1–3 receptors, but NOTCH4 is more restricted to EC. Jagged 1/Delta-like 1 (JAG1/DLL1) are mainly induced by PC, while DLL4/JAG1/2 are expressed by EC. After the generation of ligand-receptor complex, NOTCH receptor is susceptible to double cleavage by A-disintegrin and metalloprotease (ADAM) in the ECM and gamma-secretase within the cell, initiating the release of Notch intracellular domain (NICD), which translocates to the nucleus to associate with the CBF1/suppressor of hairless/LAG1 (CSL) and to switch on the transcriptional co-activator Mastermind-like (MAML), and further to activate the target genes. Instrumental for vascular homeostasis is PDGFB, chiefly secreted by EC to act on PDGFRB, expressed by PC. Sorafenib, an mTKI, inhibits VEGFR2, RAS, and PDGFRB. TGFB receptors, together with the co-receptor CD105 are evinced by the two cells, activin-like kinase 1 (ALK1) being more confined to EC. When mobilized by a ligand, type I receptors activate receptor-regulated Small Mothers Against Decapentaplegic proteins, SMADs (R-SMADs (SMAD-1, -5, and -8 for the BMP family and SMAD2 and -3 for the TGFB family)). R-SMADs connect with Co-SMAD (SMAD4) and advance to the nucleus to trigger the transcription of the genes. I-SMADs (SMAD6 for the BMP family and SMAD7 for the TGFB family) obstruct the interaction of R-SMADs with type I receptors. In ECs, ALK5 is inhibited by ALK1, so angiogenesis is promoted. The propelling of ALK5 in PC leads to the discharge of matrix metalloproteinases (MMPs) and ECM proteins like fibronectin and collagen.

### 4.2. NOTCH Signaling Pathway

The NOTCH pathway represents an evolutionary thoroughly conserved pathway that regulates fundamental cellular processes, inclusive of cell-to-cell communication, tissue differentiation, SCs maintenance, proliferation, and development, as well as cell fate ascertainment of vascular ECs and the regulation of angiogenesis [89,95,96]. The family NOTCH receptors comprises four transmembrane proteins (NOTCH1–4), present also in normal SG tissue that interrelate with five distinct sets of ligands, Delta-like (DLL-1, -3, and -4) and (Serrate-like Jagged-1 and -2 (JAG1/2)) [33,97]. The activation of the canonical pathway is appointed to double concomitant proteolytic cleavages of NOTCH receptors by two enzymes: an A-disintegrin and metalloprotease (ADAM) and gamma-secretase in the sight of Presenilin 1 and 2 (PS1/2) [98]. These contingencies lead to the release of Notch intracellular domain (NICD) [97,99,100,101] which disentangles from the plasma membrane and goes ahead to the nucleus where it connects with the transcriptional repressor CSL (CBF1/Suppressor of hairless/LAG1 or RBPJ kappa (recombining binding protein suppressor of hairless J kappa)). The NICD–RBPJ complex cooperates with a member of transcriptional co-activators such as Mastermind-like (MAML1–3) ([102]) to inaugurate the transcription of target genes like *HES1/5*, *HEY* [99], *mTORC1/2* [96,103], *PI3K* [96], *TGFB* [96], and *c-MYC* [85] to control ECs proliferation, differentiation, and apoptosis [104].

ECs mainly express DLL4, JAG1, and JAG2, in addition to NOTCH receptors 1–4, whilst PCs possess NOTCH receptors 1–3 on their surface and JAG1 and DLL1 [105,106,107,108,109]. The adjustment of cell fate commitment is mastered by NOTCH signaling and the tip/stalk cell phenotype is an emblem of the primary effect of NOTCH, too [110]. Upon ECs, VEGF brings on the generation of filopodia, attributing the supposed tip cells design [104]. The crosstalk between NOTCH and VEGF signaling are crucial for sprouting angiogenesis and for the configuration of ECs heterogeneity [111]. Guiding role of tip cells was previously reported in embryonic [112] but also adult tissues [113]. In essence, VEGF upregulates DLL4 in tip cells at the end of the sprout which further switches on Notch1 in the stalk cells, triggering the downregulation of VEGFR2 in tip cells and upregulation of VEGFR1 [104,111]. Notch signaling contributes to the prevention of excessive sprouting by the process denominated as “lateral inhibition” through activation and subduing branching of stalk cells [95,104], besides the third novel hybrid state derived from the unbalanced proportion of NICD, pivoting on divergent effects of DLL versus JAG, guiding to proliferation versus maintenance, a similar fate [114]. Although, JAG1 can contend with DLL4 through a negative feedback loop to regulate angiogenesis [95]. Moreover, the genetic deficiency of NOTCH3 correlates with the ongoing loss of PCs which associates with its performance in PCs differentiation and survival [115,116,117]. Accordingly, *NOTCH3* gain-of-function and loss-of-function denote the fact that NOTCH3 is indispensable for PCs proliferation and limitation of blood vessel permeability [109]. The unsuccessful attempt to recruit PCs to the neovessels through NOTCH signaling during angiogenesis correlates with arteriovenous malformations (AVMs), ECs hyperplasia, microaneurysms, and edemas [109]. Regarding its pivotal role in the remodeling of the vascular tree and stabilization of junctional complexes [95,110], the importance of NOTCH signaling is also substantiated in the context of atypical angiogenesis in TME [118]. Thus, it is rational to speculate that a meticulous understanding of the NOTCH signaling mechanism may lead to a new departure in the formula of cancer therapy. A lot of studies have disclosed the expression of NOTCH components in tumor contexture, especially DLL4/NOTCH1, being upregulated by VEGF [108]. For instance, inhibition of VEGF in murine neoplasms promoted the decrease of DLL4 expression, inducing non-productive vessels formation within TME, since DLL4 is required for vascular organization; therefore, concomitantly, the blockade of dual VEGF and DLL4 can bring hope to have more potent effects in tumors than the solitary obstruction of either factor [95,119,120]. Furthermore, NOTCH signaling interacts with other pathways, including MEK/ERK1/2, TGFB, AKT, and mTOR pathways [103,121,122,123]. Other reported findings impart evidence that NOTCH1 and DLL4 were overexpressed within the tumor vasculature and upregulation of *NOTCH*/c-*MYC* activates the AKT pathway via PTEN phosphorylation [123]; consequently, the NOTCH activation intensifies PI3K/mTOR activity [85,123]. In concert, the blockade of AKT directly or with PI3K inhibitors or with rapamycin (RAPA) treatment dropped off JAG1, implicating the AKT/mTOR pathway as a feedback loop in ECs [122] (Figure 2).

### 4.3. PDGF Signaling Pathway

The PDGF family of chemokines and mitogens is known to possess four members which assemble into homo- or heterodimer forms: PDGF-a, -b, -c, -d that bind to two tyrosine kinase receptors, PDGFRA and PDGFRB [124,125]. PDGFb is mainly delivered by ECs from tip cells and acts on PDGFRB, expressed by PCs [2,6,126] (Figure 2). Subsequent to new blood vessel formation, the PDGFb-PDGFRB signaling axis is paramount in PCs recruitment into the new angiogenic sprouts and vascular homeostasis [125,127]. Activation of PDGFRB upholds PC proliferation and promotes the stabilization of developing vasculature, inhibiting angiogenesis in wholly formed mature vessels [127]. KO of the *PGDFb* or *PDFGRB* genes is lethal, assignable to vascular dysfunction, and caused by PCs deficiency [128,129]. Additionally, VEGF/VEGFR2 signaling decreases PCs’ oppressive response by stimulating the release of PDGFb, and the phosphorylation of the PDGFRB, inhibiting PCs migration to the vessels undergoing active angiogenesis [130]. The complexity of the picture is sharpened by the crosstalk between PDGF, NOTCH, VEGF, and PI3K/AKT/mTOR pathways [131,132]. Loss of NOTCH signaling leads to downregulation of PDGFRB levels and PC apoptosis, showing the NOTCH regulation of PC survival and proliferation via PDGFRB [109]. Activation of PDGF is associated with the stimulation of the PI3K/AKT/mTOR pathway, while the MAPK pathway is confirmed to be unimpressed by its activation [131,133]; even so, PI3K/AKT/mTOR communicates with the MAPK pathway through signaling crosstalk [134,135]. At the administration of an AKT inhibitor, the phosphorylation of AKT was increased and downregulated mTOR, PI3K, and ERK [131]. Interestingly, the inhibition of PI3K downregulated AKT and PDGFb [132] and upregulated ERK due to the discharge of the negative regulation of AKT on the MAPK pathway [131]. Additionally, PDGF and VEGF turned down the apoptosis and increased the range of living cells in the company of the AKT inhibitor, suggesting the anti-apoptotic, pro-proliferating, and cytoprotective potential of PDGF [131]. Intriguingly, the twofold inhibitor BEZ235 of PI3K and mTOR stimulated the phosphorylation of ERK by upregulation of RAS/RAF/MEK cascade [136]. Likewise, another study reported that the inhibition of mTOR with RAPA is associated with the downregulation of VEGF and with the decrease of ECs proliferation and tumor angiogenesis [137]; thus, future studies will be required to acknowledge more specific crosstalk between these pathways in order to coin novel medical useful approaches.

### 4.4. TGFB Signaling Pathway

The TGFB superfamily members comprise more than thirty constitutional related signaling molecules, counting TGFBs *stricto sensu*, Bone morphogenetic proteins (BMPs), activin, and nodals families [138,139] that bind to several categories of receptors: TGFB receptors (TGFRBs), BMP receptors (BMPRs), and Activin-like kinases (ALKs) [140]. TGFB family emulates as the key element with pleiotropic functions in angiogenesis, migration, cell proliferation, apoptosis, and differentiation [141]. The functions of TGFB signaling have been scrupulously studied and have demonstrated the protective role in the vascular media, as well as the homeostasis and integrity [142]. The TGFB family members (TGFB1, TGFB2, and TGFB3) and BMPs signal through two major classes of Serine/Threonine kinase receptors: type I and type II [121]. Typically, when activated by a ligand, the type II receptors (TGFRBII and BMPRII) encounter a conformational change, acceding them to phosphorylate and switching on the type I receptors (TGFRBI and BMPRI) [121,140]. The type I receptors, important in angiogenesis, include ALK5, expressed on both ECs and PCs and ALK1, more restricted to ECs [6,121,140,143], and are associated with the co-receptor Endoglin [59,121]. Once activated by a ligand, type I receptors phosphorylate and, in turn, a subgroup of Small Mothers Against Decapentaplegic proteins (SMADs), the receptor-regulated SMADs (R-SMADs), including SMAD2 and -3 for TGFB family and SMAD1, -5, and -8 for BMP family, as initial responders that transduce the signal from receptors, bind to Co-SMADs (SMAD4) [143,144]. Finally, the complex R-SMAD/Co-SMAD translocates to the nucleus and regulates the transcription genes: *c-MYC*, pointed to proliferation, *HES1*, and *JAG1* [89,145,146,147]. In contrast, inhibitory SMADs (I-SMADs), including SMAD6 for the BMP family and SMAD7 for the TGFB family, are negative regulators that compete with R-SMADs to interact with activated type I receptors [148]. To dissect the specific roles of type I receptors, it should be underscored that they have opposing effects. Activation of ALK1 determines the phosphorylation of SMAD1/5/8 and promotes the proliferation of ECs and activates angiogenesis [2,149,150,151]. On the other hand, activation of ALK5 in PCs brings about phosphorylation of SMAD2/3, encouraging differentiation of PCs and the release of MMPs and ECM proteins, such as fibronectin and collagen from both PCs and ECs [152,153]. In ECs, ALK1 inhibits ALK5, suggesting the composite reciprocity, necessary for vessel development and stabilization [2] (Figure 2). Moreover, stimulation of ALK5 upregulates VEGFR1 and downregulates VEGFR2, inhibiting the proliferation of ECs [65,150]. Interestingly, it was demonstrated that TGFB collaborates with NOTCH signaling in the modulation of N-cadherin [154]. The KO of SMAD4 is related to reduced expression of N-cadherin and leads to the disruption of heterotypic contacts with PCs that further results in downregulation of TGFB signaling and disallows proliferation of PCs and promotes ECs hyperplasia [6,154]. Recent studies revealed that SMAD6, which regulates the inputs of SMAD1/5/8, as anti-angiogenic, is adjusted essentially upon NOTCH/DLL4 and VEGF levels regarding whether to promote sprouting angiogenesis or to broaden the original vasculature [110,155]. Another study delineated the alterations of TGFB1 signaling in SG pathogenesis [156]. The existence of all three isoforms of TGFB was confirmed upon ECs in patients going through SS [157]. Additionally, in mucoepidermoid carcinoma, TGFB1 was overexpressed on ECs and, of note, TGFRB2 was inversely proportional to tumor grade: low-grade tumors overexpressed TGFRBII, whereas neither high-grade tumor showed TGFRBII expression [158]. Of interest is that TGFB synchronizes ECM synthesis, along with ECs proliferation and migration, and as such TGFB1 induces PDGFb, instrumental to PCs recruitment to support stable vasculature [121].

### 4.5. HEDGEHOG Signaling Pathway

The HH signaling pathway plays an imperative role in a multiplicity of developmental and postnatal processes, including cell proliferation and differentiation, orchestrating the regulation of angiogenesis, blood vessel maturation, repair of normal tissues, and survival of normal/malignant SCs [159,160,161,162,163]. The actuating of the canonical HH pathway is denoted by the association of the three ligands-morphogens, Sonic hedgehog (SHH), Indian hedgehog (IHH), and Desert hedgehog (DHH) with the Patched1 receptor (PTCH1) and is regulated by assorted coreceptors, such as CDON, Brother of CDON (BOC), and Growth arrest specific 1 (GAS1) that promote ligand-receptor association; meanwhile, HH-interacting protein (HHIP) obstructs it [162,164]. In the absence of HH ligands, PTCH1 suppresses the activity of the transducer Smoothened (SMO) and the downstream transcription factors (TFs), GLI1, GLI2, and GLI3 are connected with Suppressor of fused (SUFU), a negative regulator of HH signaling and Kinesin family member 7 (KIF7) [162,165,166]. Notably, GLI1 constitutes the readout of the HH’s scheme, serving as the main downstream effector of the pathway, and also as a target gene [167,168,169], inasmuch as GLI2 and GLI3 organize into full-length (FL) as activator and as repressor (GLIR) configurations [170]. KIF 7 and SUFU sustain the phosphorylation of GLIFL by protein kinaseA (PKA), glycogen synthase kinase3 (GSK3), and casein kinase1(CK1) ([162,171]). Under basal conditions, the phosphorylated forms of GLI2 and GLI3 are controlled by proteasome degradation through E3 ubiquitin (UBE3) ligase complex and BTB/POZ protein/Cullin 3 (SPOP/CUL3) to induce GLI2R and GLI3R, the repressor configurations [162,172]. In contrast, the activation of HH signaling through the existence of HH ligand/receptor complex relieves the SMO inhibition that avoids the cleavage of GLI2 and GLI3 and activates the cascade of intracellular events [162], promoting the release of GLI from SUFU that translocates to the nucleus and activates HH target genes, by regulation of apoptosis (*BCL2*), cell cycle (CyclinD1(CCND1)), and *N-MYC* [162,167,173,174].

**Figure 3:**
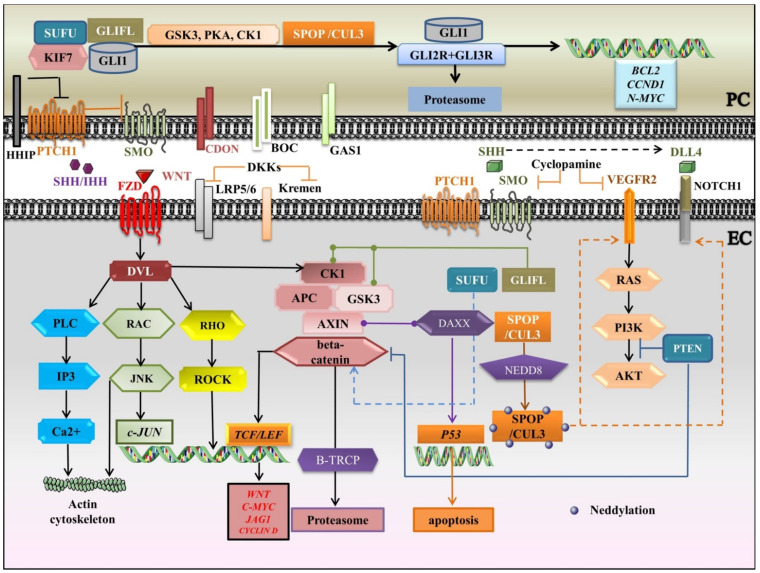
A schematic portrayal of crosstalk between HH, WNT, VEGF, and NOTCH signaling pathways. APC: Adenomatous polyposis coli; BOC: Brother of CDON; CK1: casein kinase1; CUL3: Cullin 3; DAXX: Death-domain associated protein; DKKs: Dickkopfs; DLL: Delta-like; DVL: Dishevelled; EC: endothelial cell; FZD: Frizzled receptor; GAS1: Growth arrest specific 1; GLIFL: Gli full-length; GSK3: glycogen synthase kinase-3; HHIP: HH interacting protein; HH: Indian hedgehog; IP3: Inositol 1, 4, 5-trisphosphate; JAG: Jagged; JNK: c-JUN N-terminal kinase; KIF7: Kinesin family member 7; LRP5/6: Lipoprotein receptor-related proteins 5/6; PC: pericyte; PCP: planar cellpolarity; PI3K: Phosphatidylinositol 3-kinase; PKA: protein kinaseA; PLC: Phospholipase C gamma; PTCH1: Patched1 receptor; PTM: post-translational modification; ROCK: RHO-associated protein kinase; SHH: Sonic hedgehog; SMO: Smoothened; SPOP: BTB/POZ protein; SUFU: Suppressor of fused; TCF/LEF: T-Cell factor/Lymphoid enhancing factor; UBE3: E3 ubiquitin ligase; VEGFR2: Vascular Endothelial Growth Factor Receptor 2; black arrows: main signaling pathways; blunt-ended lines: blockade/inhibition, dashed arrows: induction/activation, round-ended lines: association. Segments of the figure were sketched by using artworks from Servier Medical Art (15 November 2021). Servier Medical Art by Servier is licensed under a Creative Commons Attribution 3.0 Unported License, https://creativecommons.org/licenses/by/3.0/ (access on 15 November 2021).

Adding to this puzzle, Shh represents the most significantly expressed Hh within the vasculature, along with Ihh, expressed by ECs, and also by cancer cells in Oral squamous cell carcinomas (OSCCs) [175,176,177]. A plurality of studies sympathize in reporting the proangiogenic properties of HH ligands, especially of SHH [178,179,180]. By contrast, a genetic deficiency in SHH in murine embryos causes lethality [181]. Of note, Nielsen and Dymecki [182] delineated the angiogenic portrayal of SHH in companionship with VEGF, whereby ECs from choroid plexus induce SHH and the signal is transduced by PCs, as they expressed PTCH1 rather than their ECs counterparts, suggesting that ECs are chiefly coordinated by PCs in the throughput of HH signaling. Once again, the proangiogenic and proliferative roles of Shh should be stressed, as they promote PTCH1, GLI2, NOTCH1, NOCTH3, BCL2 in ECs, whereas they upgrade PTCH1, GLI2, and NOTCH1 in PCs [162]. GLI1 upregulates VEGFR2, as the main effector of HH-promoting angiogenesis, and HHIP in mature vessels, while HHIP is downregulated in ECs engaged in angiogenesis and tumor neovessels [162,169]. As expected, most studies emphasize the hyperactivation of HH signaling to amplify tumor angiogenesis. In detail, the inhibition of Hh signaling with cyclopamine, a SMO antagonist, decreases VEGF and PTCH1 amounts and results in the reduction of MVD in OSCC [162,183]. Furthermore, it was shown that administration of erismodegib, another SMO inhibitor, restored the MVD and reduced the PCs coverage, increasing the measure of immature vasculature [184]. Additionally, pristimerin-administered has been shown to block SHH-induced ECs proliferation and PC recruitment into neovessels, therefore inhibiting MVD and tumor growth [185]. Notably, HH signaling can integrate with elements of other major signaling pathways, including NOTCH [95], VEGF/VEGFR2 [186], and CUL3-SPOP-DAXX axis [187,188]. The crosstalk between NOTCH and SHH within the retinal microvascular compartment has been reported in vivo [175]. SHH interceded the upregulation of the NOTCH1 receptor in both PCs and ECs but was diminished after cyclopamine treatment. It was shown that high blood flow rates are accompanied by inhibition of HH and NOTCH signaling constituents that dictate the apoptosis of PCs while, interestingly, decreasing the apoptotic signals in ECs. Nonetheless, absorbing for the induction of angiogenesis is the complicity of the novel regulatory axis CUL3-SPOP-DAXX. Sakaue and colleagues [187] determined that conjugation of CUL3-based UBE3 with NEDD8, a process denominated as neddylation [189] (Figure 3), a pivotal post-translational modification (PTM) besides ubiquitination, was instrumental for the upregulation of VEGFR2, in addition to NOTCH1 and DLL4. By contrast, the knockdown of SPOP- the CUL3 substrate adaptor plus repressor of DAXX- and CUL3 generated the upregulation of Death-domain associated protein (DAXX) and downregulation of *VEGFR2* levels. Likewise, SPOP constitutes a transcriptional target of HIFs and hypoxia determines the accumulation of SPOP into the cytosol, which is satisfactory for the instauration of the tumorigenic program. The tumorigenesis materializes via ubiquitination and degradation of tumor suppressors like PTEN, ERK phosphatases, DAXX, and GLI2 [188]. Finally, by sketching the impact of HH on other pathways and by understanding the molecular mechanisms within cellular networks, a novel blueprint for the disclosure of target-discriminatory “quick-witted” drugs would revolutionize the development of medical therapy for preserving the SG function.

We summarize the crosstalk between HH, WNT, VEGF, and NOTCH signaling pathways in Figure 3. Briefly, in the HH canonical pathway, SHH/IHH-widely involved in angiogenesis- binds to PTCH1 and the inhibition of SMO is relieved, which avoids the cleavage of GLI2 and GLI3 and induces the release of GLI from SUFU and KIF7. The associated coreceptors, CDON, BOC, and GAS1 enhance ligand-receptor association, whereas HHIP inhibits it. In the absence of HH ligands, GLI1, GLI2, and GLI3 are phosphorylated by PKA, GSK3, and CK1, and are supervised by the UBE3 ligase complex, SPOP/CUL3, to induce the repressor forms, targeted for proteasome degradation. After HH ligand/receptor complex formation, the signal is mainly transduced through GLI1, serving as both downstream effector and target gene. Cyclopamine, which is a SMO antagonist, also inhibits VEGF and PTCH1 in EC. SHH upregulates the NOTCH1 receptor in both PC and EC. The neddylation (conjugation with NEDD8- a PTM) of CUL3-based UBE3 increases VEGFR2 (the main effector of HH), NOTCH1, and DLL4 levels. The WNT signaling is a key ingredient in cell proliferation/apoptosis, and vessel remodeling. WNTs associate with Frizzled (FZD), linked to co-receptors LRP5/6. Dishevelled (DVL) mediates the signal throughput to canonical and non-canonical pathways. The non-canonical WNT signaling is divided into WNT/PCP and WNT/Ca^2+^ pathways and coordinates actin cytoskeletal rearrangements. In WNT/PCP signaling, upon RAC GTPase actuating, JNK settles c-JUN transcription. In turn, RHO GTPase activates ROCK. Besides, the WNT/Ca^2+^ pathway turns on PLC, which activates IP3 to release Ca^2+^. DKKs, as WNT antagonists, associate with LRP5/6 and Kremen receptors and dictate the withdrawal of LRPs from the plasma membrane. In the canonical WNT pathway, if a WNT ligand is absent, beta catenin is phosphorylated by a destruction complex (APC, AXIN, CK1, GSK3), which is discerned by UBE3 ligase B-TRCP and targeted for proteasomal degradation, so the target genes are repressed by TCF/LEF. Once the pathway is activated by a ligand, the stabilization of beta-catenin is promoted, and it moves to the nucleus where it activates TCF/LEF and transcribes the target genes. WNT/beta-catenin collaborates with HH signaling in a positive feedback loop. The two pathways are mediated by GSK3, CK1, SUFU, PTEN, and SMO. SUFU negatively regulates GLI signaling and beta-catenin. Additionally, the loss of *PTEN* could switch on both beta catenin and GLI. Prominently, DAXX associates with AXIN to stimulate the tumor suppressor P53 to prompt apoptosis.

### 4.6. WNT Signaling Pathway

The WNT signaling pathway dictates a plethora of cellular events, including formation and remodeling of vessels, cell fate specification, proliferation, survival, and apoptosis [190,191]. The WNT family of glycoproteins bind to the Frizzled receptor (FZD), linked to the co-receptors Lipoprotein receptor-related proteins 5/6 (LRP5/6), and transduce the cellular signals to cytoplasmic phosphoprotein Dishevelled (DVL). On the level of DVL, the WNT signal splits up into substantial cascades: the canonical WNT/-beta catenin dependent pathway and the non-canonical or -beta catenin-independent pathway [190]. The non-canonical pathway is driven apart into the Planar cell polarity (PCP) and WNT/Ca^2+^ pathways [192]. The non-canonical PCP pathway switches on the small GTPases RHO and RAC and harmonizes cytoskeletal rearrangements [190]. The transduction of signal is settled through RAC activation of the c-JUN N-terminal kinase (JNK) pathway to mediate c-JUN transcription or RHO actuating of RHO-associated protein kinase (ROCK) [193,194,195]. The non-canonical WNT/Ca^2+^ pathway turns on the Phospholipase C (PLC), which in turn activates Inositol 1, 4, 5-trisphosphate (IP3) to increase intracellular Ca^2+^ [196,197,198]. Moreover, there are endogenous WNT antagonists, counting secreted rizzled-related proteins (SFRP1-5), the Dickkopfs (DKKs), the WNT inhibitory-factors (WIFs), and Cerberus [199]. DKKs connect with LRP5/6 and high-affinity receptors of the Kremen family, generating the withdrawal of LRPs from the plasma membrane [200].

The canonical WNT/- beta catenin signaling pathway governs multiple developmental events, including renewal and regeneration processes, and also the regulation of ECs growth and angiogenesis [190,201]. In the absence of WNT ligands, the cytoplasmic beta cateninis degraded via a beta catenin destruction complex which incorporates the Adenomatous polyposis coli (APC), the scaffolding protein AXIN, the Casein kinase 1 (CK1), and Glycogen synthase kinase 3 (GSK3) [196]. The phosphorylation of beta catenin by this complex induces PTMs, which is recognized by the UBE3 ligase B-TRCP and is targeted for destruction by the proteasome [202]. Together, these episodes avert the translocation of beta catenin to the nucleus and the target genes are therefore suppressed by the TFs, t-cell factor/lymphoid-enhancing factor (TCF/LEF) [196]. The pathway is activated when a canonical ligand like WNT3a, WNT4, or WNT7a/7b links to FZD and employs the DVL, which disrupts the action of the destruction complex, thereby promoting the stabilization beta catenin which moves to the nucleus [190,203]. Once there, beta catenin commutes the TCF/LEF repressor composite into a transcriptional activator system that facilitates the transcription of WNT target genes, including WNT constituents [204], *c-MYC* [196,205], *JAG1* [206], and *CCND* [196,207]. Into adulthood, WNT4 is highly expressed into SG, whereas during SG murine development, FZD-6 is upregulated constantly [201]. Notably, beta catenin interacts with N-cadherins, thus preserving their interaction with the cytoskeleton and tissue integrity [208,209]. Furthermore, in murine, the loss of beta catenin is associated with altered epithelial-mesenchymal transition (EMT) and impedes the development of the endocardial cushion [210]. The WNT/beta-catenin signaling plays a pivotal role in harmonic vascular development since deletion of beta catenin causes defective vascular remodeling and results in early lethality in utero [211]. Additionally, within the microvasculature, the WNT/beta catenin signaling controls PC recruitment [212]. WNT5a is crucial for the maintenance of post-natal homeostasis and principally activates the beta-catenin-independent WNT signaling cascade [195]. Of note, Yuan et al. [213] demonstrated that the production of non-canonical WNT5a [214] by ECs is crucial for migration of PCs toward neovessels, while WNT5a KO corresponds to reduced PCs coverage and disruption of vascular stability. Another recent study reported the crosstalk between CCN1 and PC-derived WNT5a in ECs-PCs cocultures [215]. The WNT5a signaling evoked by PCs suppresses the *CCN1* gene-a negative regulator of VEGF, in ECs, enhancing proliferation and EC hyperplasia. Prominently, WNT5a signals through FZD-ROR-RAC receptors and regulates the angiogenesis, the vascular morphogenesis via PCP, and ECs proliferation, being overexpressed in HPC/SFT [57,216]. To attest its intricacy and collusion in the vascular generation, WNT/beta-catenin cooperates with HH signaling in a positive feedback loop. Fundamentally, both pathways are adjusted by GSK3, CK1, SUFU, PTEN, and SMO [217]. SUFU represents not only a negative regulator of GLI signaling, but also it connects with β-catenin to supervise their nuclear–cytoplasmic disseminations [218]. Accordingly, loss of *PTEN* could activate both beta catenin and GLI [217]. Several studies have designated GSK3 as a convergent element among WNT/beta catenin and PI3K/PTEN signaling [219,220,221]. The accumulation of beta catenin is complemented via PTEN KO that further increases the activation of the PI3K/AKT pathway [221]. Intriguingly, *SMO* KO downregulates beta catenin levels, which is autonomous of the GLI effect [222]. On the other hand, the inhibition of HH signaling by cyclopamine reduces beta catenin [223]. Moreover, WNT/catenincatenin cooperates with NOTCH and VEGF/VEGFR2 signaling cascades. The gain-of-function of beta catenin upregulates *DLL-4* expression [191]. The depletion of *betacatenin* or *VEGFR*2 from ECs leads to hypovascularization and rescinds angiogenesis following DLL4 downregulation in tip cells [224]. The crosstalks between NOTCH and WNT pathways are interceded by the regulation of GSK3. WNT1 countermanded the phosphorylation of NOTCH2 by GSK3 which, finally led to the upregulation of HES1 [225]. Notwithstanding, another study shed light upon the mechanistic role of DAXX in regulating tumorigenicity in correlation with the beta catenin pathway. It was shown that DAXX firmly cooperates with Axin to stimulate the tumor suppressor P53 to induce apoptosis [226] (Figure 2). Thus, acknowledging the interplay between all these molecular networks can provide a platform to identify novel anti-angiogenic/tumorigenic therapies.

### 4.7. Extracellular Vesicles/Exosomes

EVs constitute a miscellaneous population of bilayer membrane nanostructures, accommodating transmembrane proteins and incorporating assorted messenger nucleic acids, counting mRNAs, microRNAs (miRNAs), also other non-coding RNAs (ncRNAs), and signaling molecules for interchanging information with recipient cells [227,228,229]. Based on their subcellular origin, EVs are organized into three subgroups: apoptotic bodies, microvesicles (MVs), and EXOs [230]. Notably, EXOs, which are the minutest subgroup of EVs, assemble as Intraluminal Vesicles (ILVs) within the endosomal compartments called Multivesicular Bodies (MVBs) and are delivered to the extracellular environment after melding of MVBs with the plasmalemma [231,232]. EXOs represent key intermediaries of cell-to-cell communication that give knowledge about the prototypical cellular background via enclosed biomolecules and surface markers [233], being available in all body fluids, including saliva [234,235]. The stereotypical detected proteins include tetraspanins (CD63, CD9, and CD81), membrane transporters (RAB GTPases), and heat shock proteins (HSP70 and HSP90) [236]. Furthermore, EXOs appear like a “double-edged sword”, given their role in manifold physiological and pathological aspects [233]. As a prognostic and diagnostic tool, salivary EXOs not only could be employed as drug conveyance vehicles, but also tumor-derived EXOs have been reported to enhance the formation of TME, accelerating angiogenesis and generating the “premetastatic niche” [237,238].

There is substantial therapeutic potential of EXOs from EC-PC communication. It was shown that activation of the HIF pathway induces an angiogenic response from PCs through the exosomal bidirectional interconnection between both cell types [36]. Of note, PC-derived EXOs (PC-EXOs) via the PTEN/AKT pathway could also improve EC potentiality to regulate blood flow and decrease HIF1A and MMP2 levels [239]. PCs inhibited PTEN expression and promoted AKT levels, reducing apoptosis of ECs [239]. The study by Yuan and colleagues [213] indicated that in a PC-EC coculture, WNT5a from EC-derived EXOs (EC-EXOs) is crucial to trigger WNT/PCP in PCs and to recruit them to pulmonary blood vessels. In contrast, WNT5a EC KO was related to pulmonary arterial hypertension and right ventricular failure, corresponding to reduced PC coverage of microvasculature. Along the same line, EC-EXOs promoted by inflammatory impetuses convey particular miRNAs that mediate responses in PCs to amplify VEGFb expression, a specific ligand of VEGFR1 [240]. Additionally, there are plenty of studies to support the crosstalk among ECs and tumor cells via EVs. OSCC-derived EVs (OSCC-EVs), containing miRNA-142-3p, can intensify TGFBRI labor in ECs, advocating angiogenesis and tumor growth [241]. Additionally, ACC-derived EXOs can downregulate beta catenin in ECs to enhance the hematogenous metastasis of ACC cells [238]. Further, epiregulin-enriched ACC-derived EXOs promote EMT by upregulating N-cadherin and downregulating E-cadherin and and GLI1 [242]. Moreover, HNC-EXOs can bolster the malignant behavior of tumor cells by the distribution of SHH, initiating the non-canonical RHO/ROCK signaling cascade, enhancing the expression of MMP9, and being positively affiliated with MVD [243]. Isolated CD146^+^ CK7^+^ alpha-SMA stromal cells in the Schneiderian membrane may be involved in EMT-related regenerative processes [244]. Considering their role as potential disease biomarkers, SGT-EXOs present dissimilarities compared with healthy individuals [245]. In detail, SGT-EXOs are greater on atomic force microscopy [245] and remarkably increase in the expression of CD63, whereas CD9 and CD81 are reduced, congruent with the standpoint that the last two surface markers can impede the neoplasm metastasis [246,247,248]. Therefore, a sympathetic comprehension of the accurate function of EVs/EXOs would supplement the prognosis appraisal and may provide novel treatment approaches for HNCs.

## 5. Salivary Pericytes, Telocytes, and Myoepithelial Cells—Putative Therapeutical Local Aids in a Brighter Future?

Congruous with the desideratum for novel therapeutic perspectives to reimpose SG function and along with the knowledge that radiation therapy brings about deleterious side effects [38], the goal is to pinpoint the regenerative potentiality of parenchymal components via self-renewal and to identify progenitor/SC populations which synchronize tissue homeostasis and regeneration [249,250].

Although several approaches aim at fundamentally regenerating the duct and acinar cell lineages, it is attractive to speculate that, in fact, restoration of microvasculature after SG damage (e.g., post-IR) is the primary target [18,22], inasmuch as blood vessels govern the homeostasis, development, metabolism, and tissue microenvironment, so angio-targeted therapeutics may rehabilitate hitherto SG disorders [251]. Within this composite microarchitecture, the performance of PCs is unassailable not only in angiogenesis but also in early development and tissue regeneration [252,253]. Intriguingly, the damaged environment draws the distinction operation as to whether PCs undergo transdifferentiation or dedifferentiation [8]. As putative multipotent SCs, PCs are considerably believed to contribute to SG restoration post-IR, in addition to the adjustment of saliva secretion both in the physiological and radio-damaged model (see [3] and references therein). Furthermore, telocytes (TCs), archetypal interstitial cells, stabilize labyrinthine companionship with both PCs and ECs through direct (nano)contacts and EXOs, along with secretory acini, exocrine epithelial ducts, nerve fibers, and SCs [254,255,256,257,258]. Additionally, TCs establish an intricate three-dimensional cellular meshwork that mediates homeostasis, remodeling, and SC activity, interestingly through electrical cytoskeletal events [259]. Conspicuously, TCs can be designated as “rulers” in supervising SCs proliferation and differentiation, regardless of their location [260]. The role of TCs in angiogenesis is well-documented in several organs during development and tissue repair [261,262]. In this regard, TCs induce VEGF and release MMP9, as well as secretory vesicles to enhance EC proliferation and directed migration [257]. Intriguingly, it has been reported that TCs mediate skeletal muscle regeneration by invading the niche of PAX7+ satellite cells and secreting VEGF [260], critical for myoblast proliferation/differentiation [263,264]. Noteworthy, in addition to double expression of PDGFRA/CD34, pivotal for TC phenotyping [265,266], they also are PDGFRB immunopositive in context-dependent localization [267,268], therefore TCs may be engaged in PC recruitment and vessel stabilization [267]. Moreover, another recent study demonstrated that treatment with miRNA-21-5p-enriched TCs-EXOs inhibited EC apoptosis and promoted the regeneration of myocardial infarction [269]. Once again, it should be stressed the regenerative potential of TCs via SC niche modulation and intercellular signaling [270,271,272]. Given these features, it has been suggested that TCs are highly involved in SG homeostasis and local immune surveillance [273] since, within minor SGs distressed by SS, TCs are preserved in periacinar areas, and are not affected by the inflammatory status [274]. Of note, a study conducted by Shoshkes-Carmel et al. [275] delineated that a TC-FOXL1+ population is thoroughly essential for SC proliferation and maintenance by induction of WNT proteins. Critically, Halpern and colleagues [276] portrayed a highly preserved population of LGR5+ TCs from intestinal villus tip niche, as a source of BMP ligands and WNT5a that orchestrates the gene expression scheme. Furthermore, it has been revealed that myoepithelial cells (MECs) act as a reserve of SCs that can proliferate and transdifferentiate to enhance regeneration, following damage of resident SCs expressing cellular plasticity [277]. Additionally, MECs are preserved through self-duplication [278]. In the adult SG, K5 expression is confined to MECs and intercalated/excretory ducts [279]. The SG plasticity may assume a changeover in cellular identity from a lineage-restricted cell to another type of differentiated cell [280]. The majority of regenerated acini in a model injury have derived from differentiated MECs and KIT+ ductal cells by retrograding toward a progenitor-like state, and thereafter re-differentiating to acinar cells [281]. Although most studies exhibit that regeneration of acinar elements, following duct ligation from an atrophic state [281], turn out mainly by self-duplication of surviving acini [282,283], it should be denoted that distinct progenitors/SCs contribute to secretory cells renewal and maintenance, too [278,284,285,286]. Interestingly, LGR5 (leucine-rich repeat-containing G-protein coupled receptor 5) represents a WNT target gene [287], and a putative SC marker [201]; thereafter, LGR5+ cells have been suggested to be part of SG SCs repertoire [288]. In adult SG, WNT/beta catenin signaling is weakly expressed but is notably turned on during effective regeneration [201]. Indeed, K5- and WNT-receptive duct cells are designated as bipotent SCs, and are able to generate both duct and acinar cells [287,289]. In contrast, K5/AXIN 2-responsive intercalated duct cells are lineage-restricted progenitor cells [279]. However, assuming that beta catenin gain-of-function promotes belligerent SG Squamous Cell Carcinomas, the proper expansion of regeneration without tumorigenesis necessitates an exquisite balance. Finally, the interplay between PCs, TCs, and MECs appears like an intricate organization with pleiotropic functions which governs SG microarchitecture, and it would be erudite to consider them as putative therapeutical targets.

## 6. Conclusions

Salivary gland (SG) microvasculature constitutes an indispensable cellular organization that possesses specialized features to maintain tissue stability and homeostasis. Pericyte-endotelial cell (PC-EC) interconnections are instrumental for vascular development, maturation, and remodeling in both physiological and pathological conditions. 

As mesenchymal stem cells (MSCs), PCs are widely explored in the field of regenerative medicine, as they represent an impregnable candidate ingredient not only for enhancing vascular integrity and angiogenesis but also to reinstitute SG function after damage, thereby accomplishing tissue regeneration and repair. The molecular events appertaining to PC-EC sophisticated interconnections were meticulously characterized to unravel the phenomena that bring about SG disorders. Biological operations, including cell proliferation, SC renewal, and differentiation, are orchestrated by a plethora of signaling pathways that cooperate with each other to harmonize the developmental and postnatal equilibrium status. Consequently, an exquisite modulation of these molecular pathways can provide a plan of action to develop novel target-selective drugs to overcome SG dysfunction.
